# Perfluorooctanesulfonate (PFOS)-induced Sertoli cell injury through a disruption of F-actin and microtubule organization is mediated by Akt1/2

**DOI:** 10.1038/s41598-017-01016-8

**Published:** 2017-04-24

**Authors:** Ying Gao, Haiqi Chen, Xiang Xiao, Wing-yee Lui, Will M. Lee, Dolores D. Mruk, C. Yan Cheng

**Affiliations:** 1 The Mary M. Wohlford Laboratory for Male Contraceptive Research, Center for Biomedical Research, Population Council, 1230 York Ave, New York, New York, 10065 USA; 2 Department of Reproductive Physiology, Zhejiang Academy of Medical Sciences, Hangzhou, 310013 China; 3 School of Biological Sciences, The University of Hong Kong, Pokfulam, Hong Kong China

## Abstract

PFOS (perfluorooctanesulfonate, or perfluorooctane sulfonic acid) is an anthropogenic fluorosurfactant widely used in consumer products. While its use in Europe, Canada and the U.S. has been banned due to its human toxicity, it continues to be used in China and other developing countries as a global pollutant. Herein, using an *in vitro* model of Sertoli cell blood-testis barrier (BTB), PFOS was found to induce Sertoli cell injury by perturbing actin cytoskeleton through changes in the spatial expression of actin regulatory proteins. Specifically, PFOS caused mis-localization of Arp3 (actin-related protein 3, a branched actin polymerization protein) and palladin (an actin bundling protein). These disruptive changes thus led to a dis-organization of F-actin across Sertoli cell cytosol, causing truncation of actin microfilament, thereby failing to support the Sertoli cell morphology and adhesion protein complexes (e.g., occludin-ZO-1, CAR-ZO-1, and N-cadherin-ß-catenin), through a down-regulation of p-Akt1-S473 and p-Akt2-S474. The use of SC79, an Akt1/2 activator, was found to block the PFOS-induced Sertoli cell injury by rescuing the PFOS-induced F-actin dis-organization. These findings thus illustrate PFOS exerts its disruptive effects on Sertoli cell function downstream through Akt1/2. As such, PFOS-induced male reproductive dysfunction can possibly be managed through an intervention on Akt1/2 expression.

## Introduction

PFOS is a global pollutant and an environmental toxicant, widely used as a fabric protector, serving as a stain repellant in drapery, carpets and clothing. Its use in consumer products has been discontinued and banned in Europe, Canada and the U.S. since the 2000s due to its health risks associated with human, wildlife and animal exposure including reduced fetal growth, endocrine disruption, reproductive dysfunction, and neonatal mortality^1–4^. However, epidemiologic evidence does not support a causal association between PFOS exposure and cancer risk in humans^5^. Nonetheless, in utero exposure to PFOS adversely affect the fetal synthesis and secretion of reproductive hormones (e.g., testosterone, estradiol, and inhibin B) in humans^6^. Since the half-life of PFOS and its related compound PFOA (perfluorooctanoic acid) is relatively long, at 5.4-yr and 3.8-yr, respectively^7, 8^, even low-dose exposure to PFOS and its related compounds can accumulate in the body over an extended period of time. At present, studies in rodents have generally supported the notion that PFOS perturbs testis function, such as by inducing Sertoli cell injury and disrupting Leydig cell steroidogenic function^9–12^. A recent study from our laboratory has shown that the PFOS-mediated Sertoli cell injury that impedes blood-testis barrier (BTB) function using an *in vitro* model of primary Sertoli cells is through a disruption of actin-based cytoskeleton in Sertoli cells, involving p-FAK-Y407^13^, which is an activated form of FAK earlier shown to be involved in BTB remodeling during spermatogenesis^14^. The notion that FAK is involved in PFOS-mediated Sertoli cell injury was further confirmed by using an endogenous miRNA specific for FAK, miR-135b, which was found to perturb the Sertoli TJ-permeability barrier alone, and also worsened PFOS-induced TJ-barrier disruption^13^. However, overexpression of a constitutive active phosphomimetic mutant of p-FAK-Y407, namely p-FAK-Y407E, by mutating Tyr(Y)-407 to Glu(E)-407, in Sertoli cells could protect these cells from the damaging effects of PFOS^13^.

Recent studies have shown that the Sertoli cell BTB function is mediated by mTORC1 complex through rpS6, involving Akt1/2 downstream, which in turn modulates F-actin organization in Sertoli cells^15^, but also involving MMP9 activation^16^. Other studies also support the involvement of FAK in mTOR signaling^17^, and the involvement of MMP2 and FAK in Akt signaling^18^. In order to better understand the signaling pathway of FAK-mediated rescue function during PFOS-induced Sertoli cell injury, we sought to examine the involvement of Akt in PFOS-mediated Sertoli cell injury, and its functional relationship with p-FAK-Y407. This mechanistic study thus provides additional insight on the molecular mechanism by which PFOS causes reproductive dysfunction in males through its disruptive effects on the Sertoli cell of the mammalian testis.

## Results

### PFOS perturbs TJ- and basal ES-protein localization in Sertoli cells – an involvement of p-Akt1/2?

**Figure 1:**
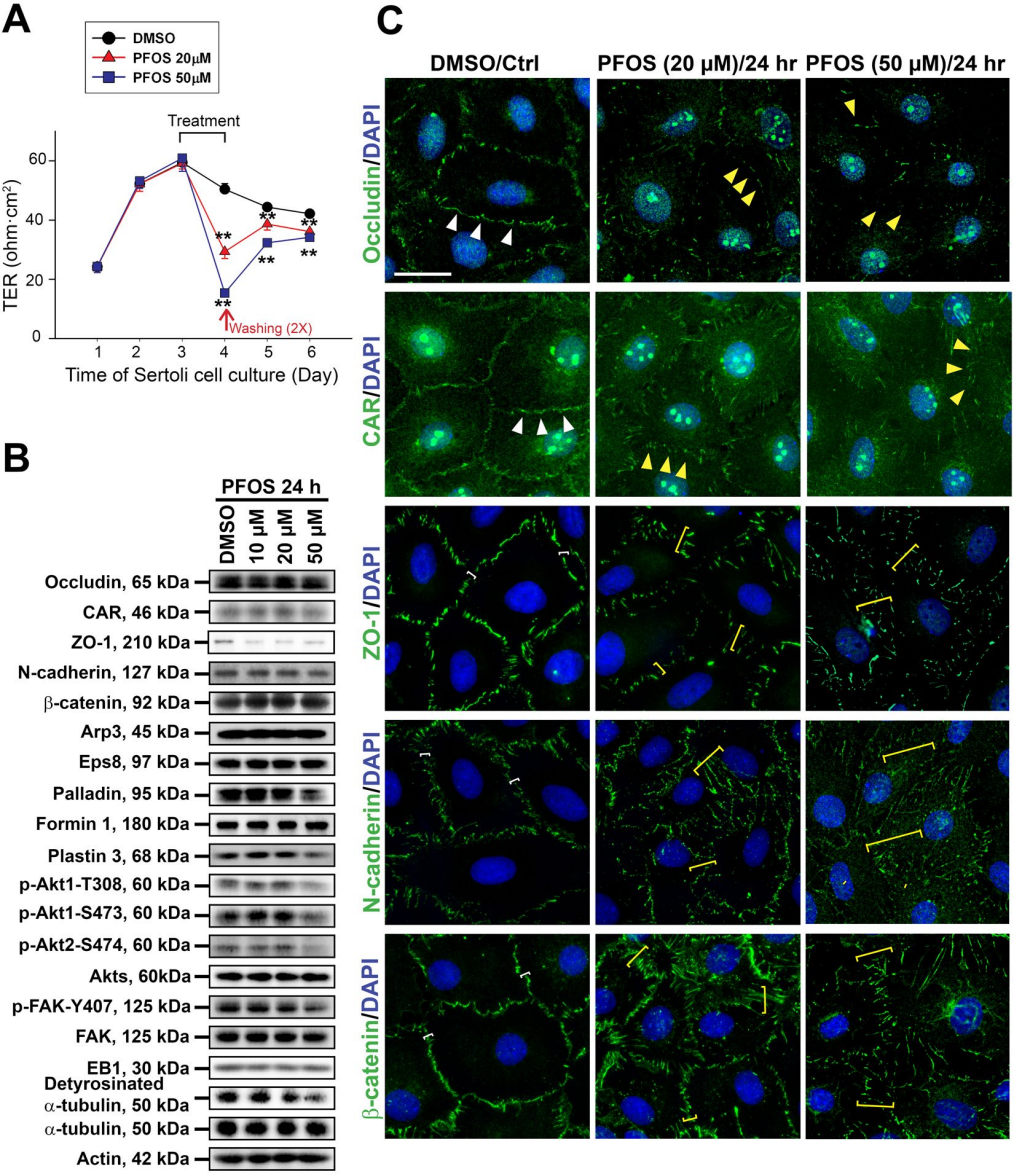
PFOS perturbs Sertoli cell BTB function through changes in the expression and/or localization of proteins at the immunological barrier. Sertoli cells cultured on Matrigel-coated bicameral units, dishes or coverslips were treated on day 3 with 10, 20 or 50 μM PFOS for 24 hr. Thereafter, cells were washed twice with PBS to remove residual PFOS and terminated for immunoblotting (IB) or IF on day 4. (**A**) Typical results of a TER experiment that monitored the effects of PFOS, at 20 µM and 50 µM *vs*. vehicle control (DMSO) on the Sertoli cell TJ-permeability barrier. Each data point is a mean ± SD of triplicate units from an experiment, which was repeated 3 times and yielded similar results. **P < 0.01 by Student’s *t*-test compared between PFOS-treated and control cells. (**B**) Immunoblot analysis to assess the effects of PFOS on the expression of TJ proteins: occludin, CAR and ZO-1; basal ES proteins: N-cadherin and β-catenin; actin regulatory proteins: Arp3, Eps8, palladin, formin 1 and plastin 3; signaling molecules: activated Akts: p-Akt1-T308, p-Akt1-S473, pAkt2-S474 *vs*. Akts (including Akt1, 2 and 3), and activated FAK: p-FAK-Tyr407 *vs*. FAK; MT regulatory proteins: EB1 and detyrosinated α-tubulin. Actin and α-tubulin served as protein loading controls. PFOS was found to down-regulate p-Akt1-T308, p-Akt1-S473, p-Akt2-S474 and p-FAK-Tyr407. Composite data of immunoblottings are shown in Figure S1A; and uncropped blots are shown in Figure S4. (**C**) Immunofluorescence analysis to assess the effects of PFOS at 20 µM and 50 µM *vs*. control on the distribution of TJ proteins: occludin, CAR and ZO-1; basal ES proteins: N-cadherin and β-catenin. PFOS caused internalization of TJ and basal ES proteins in Sertoli cells in which these proteins no longer tightly localized at the Sertoli cell-cell interface. Occludin and CAR at the cell-cell interface (annotated by white arrowheads) were diminished following PFOS treatment (yellow arrowheads). For ZO-1, N-cadherin and ß-catenin, these proteins were localized tightly at the cell-cell interface (white brackets) but were considerably internalized, diffusing away from the cell cortical zone (yellow brackets). Sertoli cell nuclei were visualized by DAPI. Composite analyzed data are shown in Figure S1B. Scale bar, 30 μm.

Sertoli cells isolated from 20-day-old rat testes were cultured *in vitro* for 3 days to form a cell epithelium with an established functional TJ-barrier, which mimicked the Sertoli cell BTB *in vivo* ^19–22^. This *in vitro*-based system has been widely used by investigators to study BTB dynamics^23–29^, and many of the findings were subsequently confirmed by studies *in vivo* ^30–33^. Thereafter, these cells were treated with PFOS at 10, 20 or 50 µM for 24 hr. It was noted that PFOS perturbed the Sertoli cell TJ-permeability barrier function dose-dependently with PFOS at 50 µM was more effectively to perturb the TJ function than at 20 µM (Fig. 1A). The range of PFOS concentration at 20–50 µM (i.e., at ~10–25 µg/ml) used in our study herein was shown not to be cytotoxic as recently reported from our laboratory, and no cytotoxicity was found at concentration up to 100 µM^13^. At present the acceptable daily intake (ADI) of PFOS is 150 ng/kg/day (for a review, see ref. 34). But since PFOS has a human elimination half-life of 5.4-year^7, 8^, a considerable level of PFOS could build up in humans in particular among industrial workers such as those who utilize PFOS for manufacturing of stain resistant carpets and fabrics.

Lysates from Sertoli cells at specified time points were then obtained for immunoblotting using corresponding specific antibodies which were characterized earlier in our laboratory (Table 1)^35, 36^. The steady-state levels of TJ-proteins occludin and CAR, and basal ES-proteins N-cadherin and ß-catenin, branched actin polymerization protein Arp3, barbed end capping and bundling protein Eps8, and actin nucleation protein formin 1 were not perturbed following PFOS treatment, but TJ-adaptor protein ZO-1 and actin bundling proteins palladin and plastin 3 were down-regulated (Fig. 1B; Figure S1A). More important, considerable down-regulation of p-Akt1-T308, p-Akt1-S473, and p-Akt2-S474 were noted, but not total Akts including 1–3 (Fig. 1B; Figure S1A). The down-regulation of p-FAK-Y407, but not total FAK, was also noted (Fig. 1B), consistent with an earlier report^13^. We also detected no changes in the expression EB1 (a plus (+)-end tracking protein, +TIP, of MT, known to stabilize MT^37^) but a down-regulation on the expression of detyrosinated α-tubulin (i.e., removal of C-terminal Tyr by exposing Glu as the N-terminal residue known to stabilize MT, making MT less dynamic^38^). Gross mis-localization of TJ proteins occludin, CAR and ZO-1, as well as basal ES proteins N-cadherin and ß-catenin at the Sertoli cell-cell interface was also noted wherein these proteins no longer tightly associated with the cell cortical zone, but internalized and moved into the cell cytosol (Fig. 1C; Figure S1B). These changes thus de-stabilized the Sertoli cell barrier function.

**Table 1:** Antibodies used for different experiments in this study.

Antibody	Host species	Vendor	Catalog no.	Working dilution	
				IB	IF/IHC
Actin	Goat	Santa Cruz Biotechnology	sc-1616	1:300	
Arp3	Mouse	Sigma-Aldrich	A5979	1:3000	1:50
Akt	Rabbit	Cell Signaling Technology	9272	1:1000	
p-Akt T308	Rabbit	Cell Signaling Technology	2965	1:1000	
p-Akt1 S473	Rabbit	Cell Signaling Technology	4060	1:1000	
p-Akt2 S474	Rabbit	Cell Signaling Technology	8599	1:1000	
α-tubulin	Mouse	Abcam	ab7291		1:500 (tissue) 1:300 (cell)
β-catenin	Rabbit	Thermo Fisher Scientific	71–2700	1:2000	1:100
CAR	Rabbit	Santa Cruz Biotechnology	sc-15405	1:200	1:50
Detyrosinated α-tubulin	Rabbit	Abcam	ab48389	1:1000	1:200
EB1	Mouse	BD Biosciences	610534		1:200
EB1	Rabbit	Santa Cruz Biotechnology	sc-15347	1:200	
Eps8	Mouse	BD Biosciences	610143	1:5000	1:50
Formin 1	Mouse	Abcam	ab68058	1:500	1:50
FAK	Mouse	Millipore	05–537	1:1000	
p-FAK-Tyr407	Rabbit	Thermo Fisher Scientific	44–650G	1:1000	1:50
N-cadherin	Mouse	Thermo Fisher Scientific	33–3900	1:200	1:100
Occludin	Rabbit	Thermo Fisher Scientific	71–1500	1:250	1:100
Palladin	Rabbit	Proteintech	10853–1-AP	1:1000	1:100
Plastin 3	Rabbit	Abcam	ab137585	1:500	
ZO-1	Rabbit	Thermo Fisher Scientific	61–7300	1:250	1:100
Goat IgG-HRP	Bovine	Santa Cruz Biotechnology	sc-2350	1:3000	
Rabbit IgG-HRP	Bovine	Santa Cruz Biotechnology	sc-2370	1:3000	
Mouse IgG-HRP	Bovine	Santa Cruz Biotechnology	sc-2371	1:3000	
Rabbit IgG-Alexa Fluor 488	Goat	Thermo Fisher Scientific	A-11034		1:250
Mouse IgG-Alexa Fluor 488	Goat	Thermo Fisher Scientific	A-11029		1:250

Abcam, Cambridge, MA; Cell Signaling Technology, Danvers, MA; Santa Cruz Biotechnology, Santa Cruz, CA; Sigma-Aldrich, St. Louis, MO; Invitrogen, Life Technologies, Carlsbad, CA; Proteintech, Chicago, IL; BD Biosciences, San Jose, CA; Millipore Corp., Billerica, MA.

### PFOS perturbs Sertoli cell TJ-barrier function through changes in the spatial expression of actin binding and regulatory proteins

**Figure 2:**
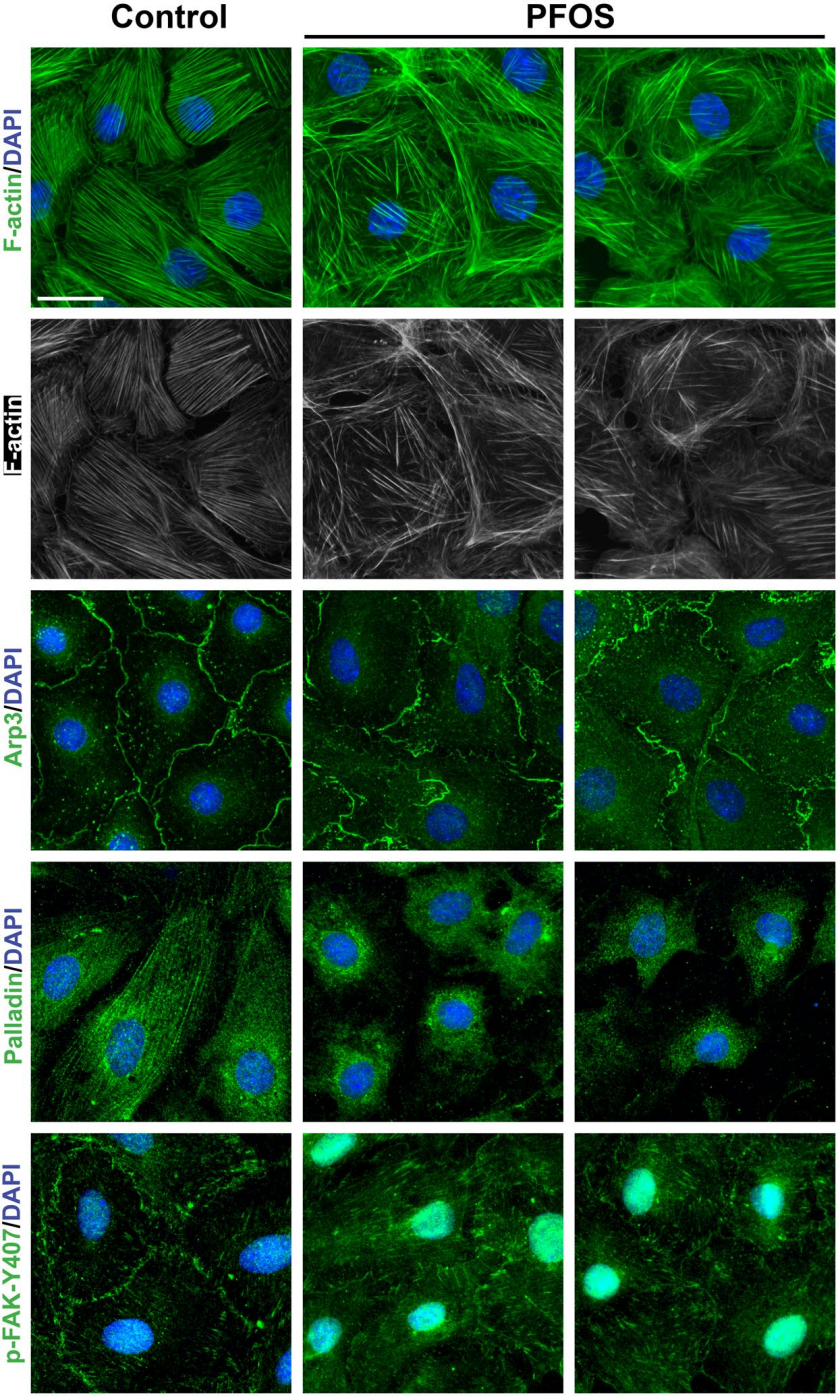
Immunofluorescence analysis to assess the effects of PFOS on the organization of actin microfilaments in Sertoli cells via changes on the distribution of actin regulatory proteins. Sertoli cells cultured for 3 days were treated with 20 μM PFOS for 24 hr. Cells were then harvested and used for IF, and results from two different experiments were shown in middle and right columns *vs*. the control cells in the left column. PFOS caused truncation and defragmentation of F-actin network (green and gray). These changes in the organization of actin microfilaments were likely the result of changes in the spatial localization of several actin regulatory proteins. Arp3, a branched actin polymerization protein that effectively converted actin microfilaments into a branched/unbundled configuration no longer localized at the cell-cell interface to support F-actin dynamics at the cell cortical zone. Instead, Arp3 was internalized and found in the Sertoli cell cytosol, thereby impeding actin microfilaments to organize as long actin bundles stretched across the cell cytosol. Palladin, an actin cross-linking and bundling protein, no longer stretched across the Sertoli cell cytosol to maintain actin microfilaments across the Sertoli cells as found in control cells. Instead, palladin was retracted from the cell peripheries but concentrated to cell nuclei after PFOS treatment. p-FAK-Y407, a signaling protein shown to be involved in maintaining BTB integrity by maintaining F-actin network in Sertoli cells as actin filament bundles also no longer localized to the Sertoli cell-cell interface to maintain the TJ-barrier function. Instead, it was internalized into the cell cytosol, thereby destabilizing the TJ-barrier function. Sertoli cell nuclei were visualized by DAPI. Scale bar, 30 μm.

Since the TJ- and basal ES-proteins that displayed mis-localization at the Sertoli cell-cell interface noted in Fig. 1C all utilized F-actin for attachment, we next examined any changes in the F-actin organization in Sertoli cells following PFOS treatment. As expected, F-actin organization was grossly affected by PFOS treatment in which long stretches of actin microfilaments no longer laid across the Sertoli cell cytosol in an organized fashion as noted in control cells (left column), they became truncated, and in some cells they were also branched in two different experiments shown in the middle and right columns (Fig. 2). These changes in actin organization appeared to be the result of changes in spatial localization of branched actin polymerization protein Arp3 and actin bundling protein palladin which confer actin microfilaments into a bundled configuration, leading to actin filament bundles into a disorganized branched network. For instance, Arp3 no longer localized mostly at the cell-cell interface, but re-localized and moved into the cell cytosol (Fig. 2). Palladin also no longer stretched across the Sertoli cell cytosol to organize actin filaments into bundles as seen in controls, instead, paladin retracted from cell cytosol and found closer to the cell nuclei following PFOS treatment (Fig. 2). p-FAK-Y407, a known TJ regulator in the testis^14^ also considerably mis-localized in which they no longer found at the Sertoli cell-cell interface, but all diffusely localized in the cell cytosol (Fig. 2).

### Rescue of PFOS-induced Sertoli cell TJ-barrier disruption by SC79, an activator of Akts through changes in the re-distribution of BTB-associated proteins

**Figure 3:**
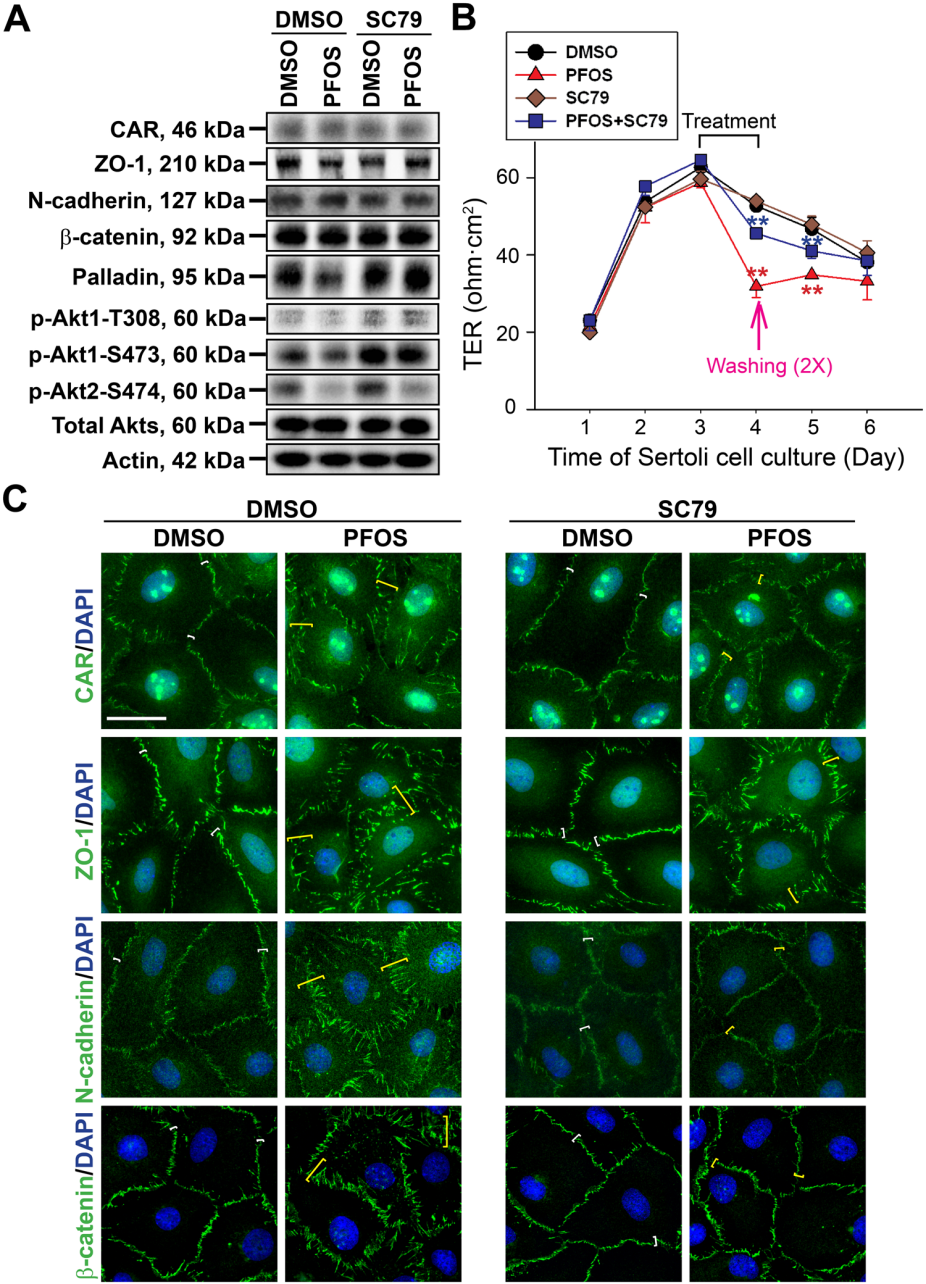
Akt activator SC79 blocks PFOS-mediated Sertoli cell TJ-barrier disruption and mis-localization of TJ and basal ES proteins at the BTB. (**A**) Sertoli cells cultured at 0.1 × 10^6^ cells/cm^2^ (in 6-well dishes with 2 ml F12/DMEM per well) for 3 days were pre-treated with SC79 at 2 μg/ml (5.5 µM) for 30 min. Cells were rinsed and treated with 50 μM PFOS for 24 hr for immunoblotting. SC79 was found to up-regulate the PFOS-induced down-regulation of p-Akt1 T308 and p-Akt1 S473, but had no apparent effect in blocking the PFOS-induced down regulation of p-Akt2 S474. SC79 was also found to rescue PFOS-induced down-regulation on the expression of ZO-1, palladin. Actin served as a loading control. Composite analyzed data of immunoblottings are shown in Figure S2A; and uncropped blots are shown in Figure S5. (**B**) A study by TER to assess the ability of SC79 to block the PFOS-mediated disruption of the Sertoli cell TJ-permeability barrier function. Treatment of PFOS alone perturbed the TJ-barrier function, **P < 0.01, by Student’s *t*-test compared between PFOS treated cells *vs*. control (DMSO) cells; **P < 0.01, by Student’s *t*-test compared between PFOS + SC79-treated cells vs. PFOS-treated cells. (**C**) Sertoli cells cultured at 0.04 × 10^6^ cells/cm^2^ for 3 days were pre-treated with 2 μg/ml SC79 for 30 min, and then cells were rinsed and treated with 20 μM PFOS for 24 hr. Thereafter, cells were then fixed and examined by immunofluorescence microscopy. SC79 treatment was found to block PFOS-induced mis-localization of TJ proteins CAR and ZO-1, as well as basal ES proteins N-cadherin and β-catenin. BTB-associated proteins at the Sertoli cell-cell interface in control and PFOS-treated cells were annotated by corresponding white and yellow brackets, respectively. Composite analyzed data of this experiment are shown in Figure S2B. Sertoli cell nuclei were visualized by DAPI. Scale bar, 30 μm.

Recent studies have shown that Sertoli cell TJ-barrier disruption is mediated downstream via Akt1/2 through a down-regulation of p-Akt1-S473 and p-Akt2-S474^15^. We speculated an induction of p-Akt1/2 via the use of a membrane permeable Akt phosphorylation activator SC79^39^ might prevent the PFOS-mediated Sertoli cell TJ-permeability disruption, and might also rescue the PFOS-induced mis-localization of TJ- and basal ES-proteins at the Sertoli cell-cell interface. Indeed, the use of SC79 was found to block the PFOS-induced down-regulation of ZO-1, palladin, p-Akt1-T308 and p-Akt1-S473 (but not p-Akt2-S474) (Fig. 3A; Figure S2A). It is of interest to note that SC79 itself activated p-Akt1-T308 and p-Akt1-S473 in DMSO-vehicle control group (Fig. 3A **lane 1** ***vs***. **lane 3**). However, SC79 *per se* had no apparent effects on the Sertoli cell TJ-permeability barrier function (Fig. 3B). But SC79 blocked the PFOS-induced Sertoli cell TJ barrier disruption when the barrier function was monitored across the Sertoli cell epithelium on Matrigel-coated bicameral units (Fig. 3B). The ability of SC79 to rescue Sertoli cells from the disruptive effects of PFOS onto the TJ-barrier function was mediated through changes in the re-distribution of TJ-proteins CAR and ZO-1, as well as basal ES-proteins N-cadherin and β-catenin since SC79 treatment managed to re-localize these BTB-associated proteins back to the Sertoli cell-cell interface compared to PFOS-treated cells without SC79 pre-treatment (Fig. 3C; Figure S2B). These findings thus support the notion that PFOS exerts its disruptive effects on the Sertoli cell-TJ barrier function via p-Akt signaling protein involving p-Akt1-T308 and p-Akt1-S473 but not p-Akt2-S474. Furthermore, this disruptive effect can be blocked via the use of an Akt activator SC79.

### Rescue of PFOS-induced Sertoli cell TJ-barrier disruption by SC79 through changes in the organization of actin-based cytoskeleton

**Figure 4:**
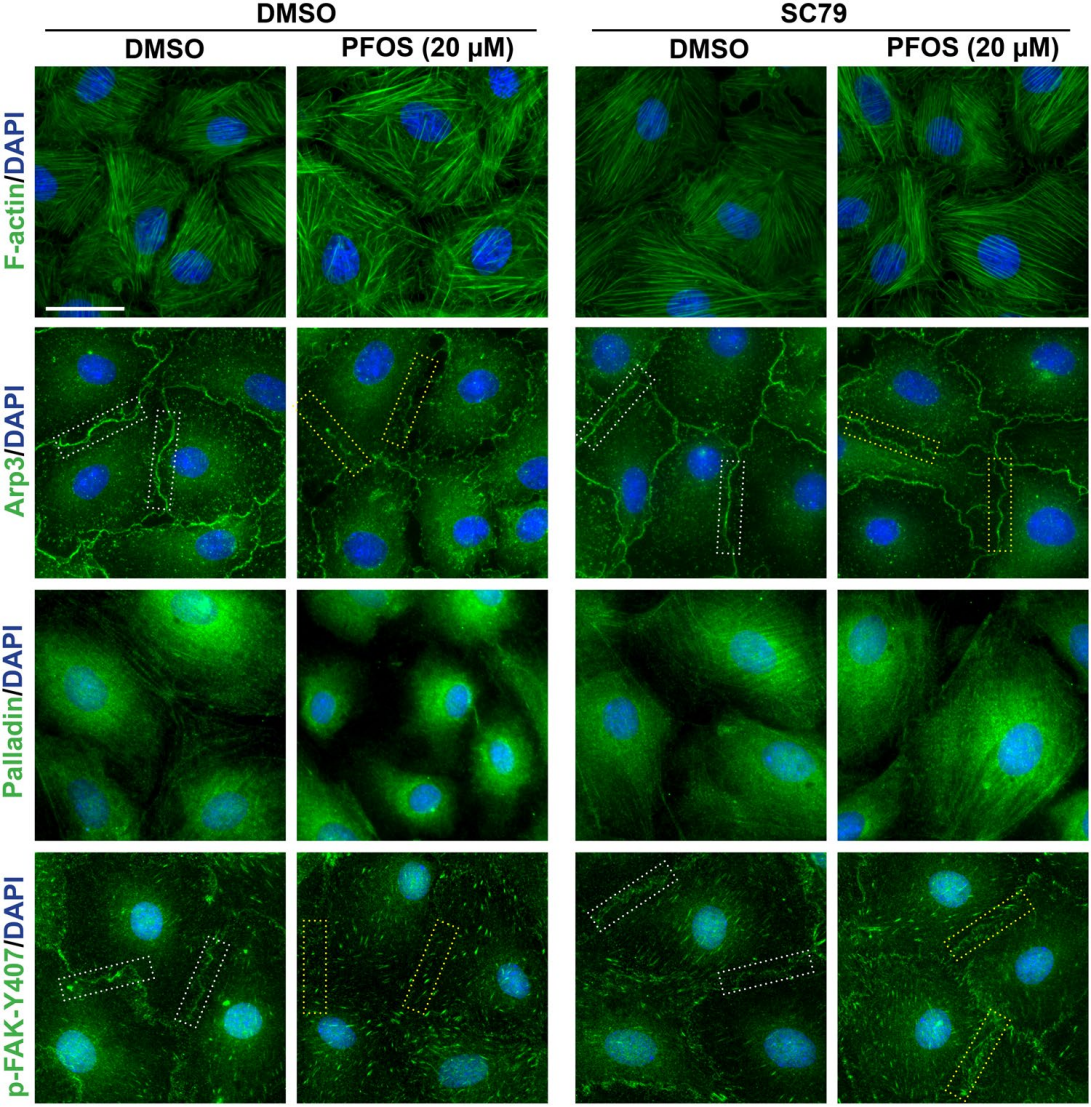
SC79 blocks PFOS-induced disorganization of actin microfilaments by maintaining proper spatial expression of actin regulatory proteins in Sertoli cells exposed to PFOS. Sertoli cells cultured at 0.03 × 10^6^ cells/cm^2^ for 3 days were pre-treated with 2 μg/ml SC79 (5.5 µM) for 30 min, and then cells were rinsed and treated with 20 μM PFOS for 24 hr. Cells were then fixed and visualized by immunofluorescence microscopy. It was noted that Sertoli cells exposed to PFOS without pre-treatment with SC79 were grossly disorganized in which actin microfilaments were truncated, likely the result of changes in the spatial expression of Arp3 (a branched actin polymerization protein), palladin (an actin cross-linking and bundling protein) and p-FAK-Y407 (a BTB-associated signaling protein known to promote BTB integrity^14^). However, pretreatment of Sertoli cells with SC79 blocked the PFOS-induced disorganization of actin microfilaments, causing actin microfilaments to be organized similar to control cells even in the presence of PFOS for 24 hr. This blocking effect of SC79 on PFOS-induced Sertoli cell injury apparently was mediated by maintaining the proper spatial expression of Arp3, palladin and p-FAK-Y407 as noted in control cells. Distribution of the target proteins Arp3 and p-FAK-Y407 at the cell cortical zone was also quantified by measuring the fluorescence intensity at the Sertoli cell-cell interface (see dotted white rectangles in control cells *vs*. dotted yellow rectangles in PFOS-treated cells), which was the average of 4 measurements (i.e., fluorescence intensity) at two opposite ends of a Sertoli cell as annotated by the rectangles for each cell. About 70 randomly selected cells in each experiment were quantified with *n* = 3 independent experiments. Scale bar, 30 μm, which applies to all other micrographs.

We next examined the underlying molecular mechanism by which SC79, the activator of Akt, blocked the PFOS-mediated disruption on the Sertoli cell TJ-permeability barrier and by re-distributing BTB-associated proteins at the Sertoli cell-cell interface as noted in Fig. 3. As shown in Fig. 4 (top left panel), treatment of Sertoli cells with PFOS induced disorganization of actin microfilaments in Sertoli cells through changes in the spatial expression of Arp3, palladin and p-FAK-Y407 (a BTB regulator that confers BTB integrity) (Fig. 4, **left panels**). For instance, actin microfilaments across the Sertoli cell cytosol became truncated, no longer orderly organized and that stretched across the cell cytosol as found in control cells treated with DMSO alone (Fig. 4). The orderly alignment of actin microfilaments found in control cells were likely the result of spatial expression of Arp3 and p-FAK-Y407, mostly at the cell-cell interface and also palladin which conferred actin microfilaments their bundled configuration as noted in control cells (Fig. 4). Following PFOS treatment, Arp3 no longer localized prominently at the cell-cell interface but internalized, similar to p-FAK-Y407 (**see** Figure S3), and palladin appeared to be retracted from the cell cytosol but localized closely to the cell nucleus (Fig. 4). However, treatment of SC79 prevented PFOS-induced changes in the spatial expression of Arp3 wherein this branched actin polymerization protein became localized more prominent at the Sertoli cell-cell interface, similar to control, instead of rapidly internalized as noted in PFOS-treated Sertoli cells (Fig. 4, Figure S3). This pattern of changes was similar to p-FAK-Y407 (Fig. 4, Figure S3). Furthermore, palladin was also found to stretch across the entire Sertoli cell cytosol in SC79 treated cells, blocking the PFOS-induced disruption of palladin distribution across the cell cytosol as found in PFOS-treated cells alone (Fig. 4). These changes thus contributed to the re-organization of actin microfilaments so that SC79 rescued PFOS-mediated F-actin disorganization (Fig. 4).

### PFOS dose-dependently mediates disorganization of microtubules (MTs)

**Figure 5:**
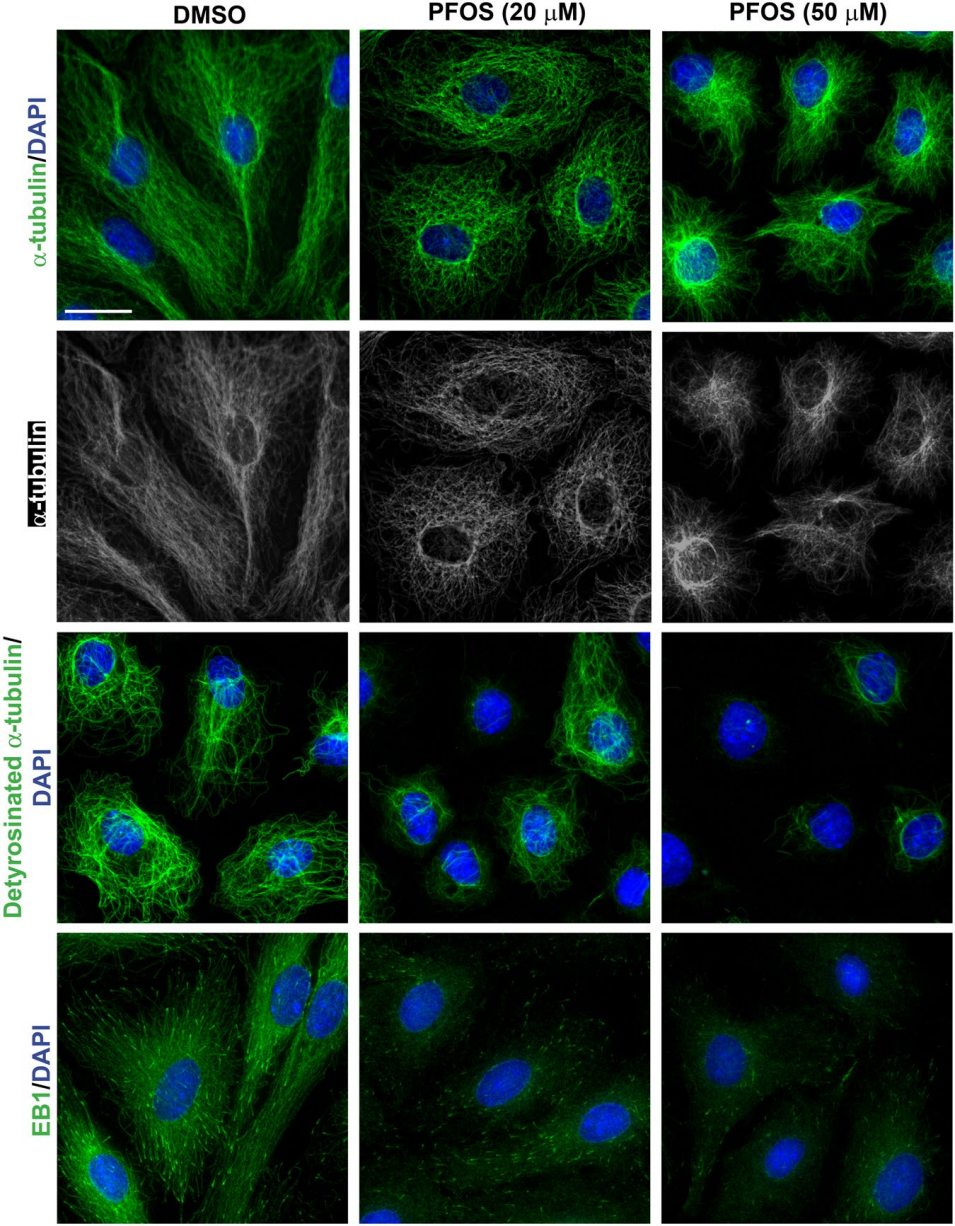
PFOS induces disorganization of microtubules (MTs) through its disruptive effects on the localization of α-tubulin and detyrosinated α-tubulin, and MT regulatory protein EB1. Sertoli cells cultured for 3 days at a density of 0.03 × 10^6^ cells/cm^2^. Cells were then treated with either 20 or 50 μM PFOS for 24 hr. Thereafter, cells were fixed with ice-cold methanol and visualized by immunofluorescence microscopy. In control cells, α-tubulin, the building block of MTs, stretched across the entire Sertoli cell to support cell shape and served as tracks for intracellular transport and trafficking. Following PFOS treatment, there was a dose-dependent retraction of MTs across the cell cytosol wherein MTs no longer stretched across the cytosol but encircled the Sertoli cell nuclei instead. Detyrosinated α-tubulin, the stabilized form of MTs, was considerably diminished dose-dependently, consistent with findings shown in Fig. 1B. Interestingly, the spatial expression of EB1, a +TIP (plus-end tracking protein) was considerably disrupted since it no longer expressed prominently across the MTs as noted in control Sertoli cells, but apparently dispersed in the cell cytosol since the overall expression of EB1 was not shown to be down-regulated as noted by immunoblotting shown in Fig. 1B. Sertoli cell nuclei were visualized by DAPI. Scale bar, 30 μm, which applies to all other micrographs.

Treatment of Sertoli cells with PFOS was found to cause disorganization of MTs when α-tubulins (the building blocks of MTs) across the Sertoli cell cytosol were visualized by IF (Fig. 5). For instance, MTs no longer stretched across the cell cytosol as found in control cells (DMSO alone) but retracted closer to the cell nuclei when PFOS was used at 20 µM. At 50 µM, even more MTs were retracted to the cell nuclei (Fig. 5). Furthermore, detyrosinated α-tubulin (the stabilized form of MTs) no longer radiated from the Sertoli cell nuclei but retracted closer to nuclei when exposed to PFOS at 20 µM, and considerably diminished more at 50 µM (Fig. 5), illustrating the dose-dependent disruptive effects of PFOS, consistent with the IB findings shown in Fig. 1B. Interestingly, the +TIP (plus-end tracking protein) EB1 (known to stabilize MTs) also no longer stretched across the cell cytosol by forming aggregates along the Sertoli cell MTs as noted in control (DMSO only) cells, but possibly dispersed singularly instead of aggregates so that the green fluorescence appeared to be weakened (Fig. 5) since no down-regulation of EB1 was noted following PFOS treatment in Sertoli cells (**see** Fig. 1B).

### SC79 recues PFOS-mediated disruption of MT organization through changes in detyrosinated α-tubulin and EB1 distribution

**Figure 6:**
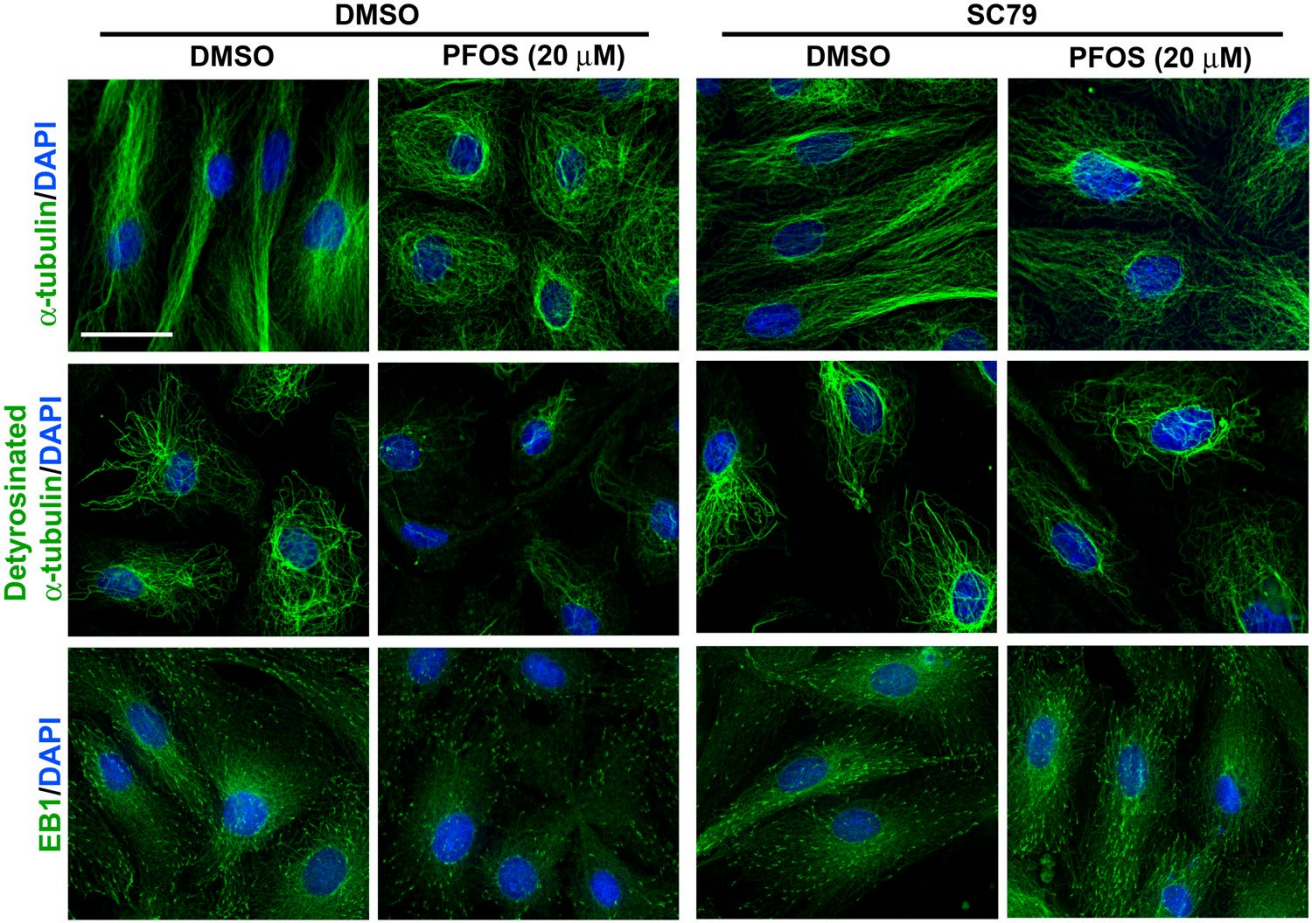
Akt activator SC79 blocks PFOS-mediated MT (e.g., α-tubulin and detyrosinated α-tubulin) disorganization by attenuating disruptive distribution of EB1. Sertoli cells cultured for 3 days at a density of 0.03 × 10^6^ cells/cm^2^. Cells were pre-treated with 2 μg/ml SC79 (5.5 µM) for 30 min, and then cells were rinsed and treated with PFOS at 20 µM for 24 hr. Thereafter, cells were fixed with ice-cold methanol and visualized by immunofluorescence microscopy using corresponding antibodies (Table 1). Consistent with data shown in Fig. 5, PFOS induced disorganization of MTs by disrupting the organization of α-tubulin and detyrosinated α-tubulin across the Sertoli cell cytosol and also +TIP EB1. However, SC79 blocked the PFOS-mediated MT disorganization, possibly by attenuating disruptive distribution of EB1 in SC79 pre-treated cells. Sertoli cell nuclei were visualized by DAPI. Scale bar, 30 µm, which applies to all other micrographs.

Treatment of Sertoli cells with SC79 also rescued PFOS-induced disruption of MT organization, in which MTs were found to stretch across the entire cell cytosol from cell nuclei instead of retracting from cell peripheries to form round-shaped cells in cells treated with PFOS (20 µM) without SC79 (Fig. 6, **top panel**). This protective effect of SC79 appeared to be mediated by re-distributing detyrosinated α-tubulin (a stabilized form of MTs) in the Sertoli cell cytosol, so that detyrosinated α-tubulin that was retracted from the cell cytosol in PFOS-treated cells was found to radiate from the Sertoli cell nucleus and stretches further into the cell cytosol in SC79 pretreated cells (Fig. 6, **second panel**). Similarly, EB1, a +TIP, also stretched across the Sertoli cell cytosol following SC79 treatment, by preventing PFOS-induced mis-localization of EB1, in which EB1 formed aggregates along the MTs to induce MT stabilization in SC79-treated cells even with PFOS exposure, considerably different from Sertoli cells treated with PFOS alone (Fig. 6, **bottom panel**).

## Discussion

Findings reported herein are consistent with other data in the literature that cytoskeletons in Sertoli cells, including both the F-actin and the microtubule (MT)-based cytoskeletons, are one of the targets of environmental toxicants (for reviews, see refs 40–45). Besides Sertoli cells isolated from rodent testes as noted herein, F-actin cytoskeleton in human Sertoli cells is also highly susceptible to environment toxicants, such as cadmium chloride (CdCl_2_) and bisphenol A (BPA), which were shown to induce defragmentation of actin microfilaments in Sertoli cells, thereby perturbing the distribution of TJ proteins (e.g., ZO-1) and basal ES proteins (e.g., N-cadherin, ß-catenin) at the Sertoli cell-cell interface^46^. In this context, it is of interest to note that CdCl_2_ remains widely used in the United States, such as manufacturing of batteries, and contaminating our environment through municipal waste incineration, plus an increase in human uptakes through contaminated water and food supplies including cigarette smoking; whereas BPA is found in many household items (e.g., utensils, plastics, food containers) (for BPA) (for reviews, see refs 34, 47). While the elimination half-life of BPA is short in humans (<2 hr), but cadmium has a half-life of >20 years so that considerable amount of toxic level of cadmium can build up in humans over time. Thus, the observation that human Sertoli cells are susceptible to these toxicants is of interest since these findings reproduce some of the earlier observations in studies using rodent Sertoli cells, illustrating F-actin organization is one of the targets of toxicants^13, 48^. This unexpected disruption of the F-actin organization in Sertoli cells induced by toxicants such as PFOS as illustrated herein vs. cadmium and BPA shown earlier is of physiologically important, since these findings implicate that the BTB function is compromised in the testis *in vivo* following exposure to toxicants as noted earlier^49–53^. This also explains earlier observations that the testis is highly vulnerable to toxicants (e.g., cadmium) as reported six decades ago^54, 55^. Indeed, subsequent studies confirmed the BTB was compromised before changes in the permeability of the microvessels in the testis^49^ when the kinetics of their disruption were assessed^56^, illustrating while the BTB is one of the tightest blood-tissue barriers (for reviews, see refs 20, 57), it is highly susceptible to environmental toxicants when compared to other blood-tissue barriers such as the endothelial TJ-barrier. Studies in recent years have also cautioned a declining trend of semen quality and/or sperm counts in men is associated with an increase in environmental toxicant exposure^58, 59^. Perhaps, this is due to the readily disrupted BTB, this thus increases the bioavailability of the toxicant in the testis behind the BTB, exposing post-meiotic spermatids to the toxicant.

Thus, it is important to identify the signaling molecule(s) mediated by toxicants through which the BTB can be modulated. Akt (also known as PKB) is a Ser/Thr protein kinase composed of three closely related and widely expressed isoforms of Akt1, Akt2 and Akt3 found in virtually all mammalian cells (for reviews, see refs 60, 61) including Sertoli cells^21^. In fact, Akt/PKB is prominently expressed at the ES (a testis-specific actin-rich anchoring junction found at the Sertoli cell-cell interface at the BTB known as basal ES *vs*. the similar ultrastructure found at the Sertoli cell-spermatid interface known as apical ES in the adluminal (apical) compartment of the seminiferous epithelium (for reviews, see refs 62, 63)) in the adult rat testis^21^. As reported herein, Akt1/2 was shown to be a putative target of PFOS. First, treatment of Sertoli cells with an established functional TJ-barrier was found to perturb the barrier function, associated with a down-regulation on the expression of p-Akt1-T308, p-Akt1-S473, and p-Akt2-S474. Second, the use of SC79, an Akt activator, was found to restore the steady-state protein level of p-Akt1-T308 and p-Akt1-S473, but had no apparent effect on p-Akt2-S474. These findings thus illustrate that PFOS-induced p-Akt2-S474 down-regulation could not be rescued by SC79, and this down-regulation had no correlation with PFOS-induced Sertoli cell TJ-barrier disruption nor the underlying cytoskeletal disorganization. It is known that p-Akt1-T308 and p-Akt1-S473 are working in concert to modulate cellular functions^64^. In fact, modification of Akt1 at T308 and S473 via phosphorylation has been established as the equivalent of Akt activation, illustrating these two sites are working in concert to modulate cellular function (for a review, see ref. 60). Thus, our findings that PFOS-induced p-Akt1-T308 and p-Akt1-S473 inactivation via their down-regulation, and a restoration of the leaky BTB by SC79 through an up-regulation of Akt1 at these two sites are consistent with the earlier reports^60, 65^. An interesting observation in this report is the finding that the use of SC79 was shown to induce re-organization of both F-actin- and MT-based cytoskeletons, making the PFOS-treated cells more similar to control cells, blocking the disruptive effects of PFOS on the organization of actin microfilaments and MTs. Since all the TJ- and basal ES-proteins are actin-based adhesion protein complexes, the re-establishment of the F-actin cytoskeleton thus provides the platform for re-distribution of these BTB-associated proteins so that the TJ-barrier function can be restored.

In this context, it is of interest to note that treatment of Sertoli cells with SC79 alone was found to up-regulate p-Akt1-T308 and p-Akt1-S473 (but not p-Akt2/S474), consistent with the concept that p-Akt1-T308 and p-Akt1-S473 are working together to regulate cell function^64^, yet SC79 alone had no apparent effects on the Sertoli cell TJ-permeability barrier. These observations thus suggest that there are other proteins and/or signaling pathways involved in modulating the SC79-mediated rescue efforts following PFOS exposure regarding re-organization of the actin- and MT-based cytoskeletons. This notion is supported by studies demonstrating that overexpression of connexin 43 (Cx43, a gap junction protein highly expressed in the testis^66, 67^ and a target of environmental toxicant^67^) also intercepts the PFOS-induced BTB disruption and capable of rescuing PFOS-mediated F-actin disorganization^36^. For instance, in the absence of PFOS, Cx43 *per se* that is known to maintain the Sertoli cell TJ-permeability barrier function^68, 69^ might have conferred the TJ-barrier function to its maximal, thus, the presence of SC79, including the activation of p-Akt1-T308 and p-Akt1-S473 could no longer enhance the immunological barrier to make it “tighter”. This possibility, however, must be carefully evaluated in future studies.

Earlier studies have shown that FAK, in particular p-FAK-Y407 is an important signaling molecule tightly involved in BTB integrity, playing a role in the organization of actin-based cytoskeleton in Sertoli cells^14, 70^. Studies in other mammalian cells have shown that FAK is the upstream signaling protein of Akt^71, 72^, and these two signaling proteins often work as close partners to modulate a number of cellular functions in particular tumorigenesis^73^. In fact, we also report herein the use of SC79 could restore the spatial expression of p-FAK-Y407 in Sertoli cells disrupted by PFOS, suggesting that these two functional signaling kinases may indeed work together to modulate Sertoli cell BTB function. This possibility should be carefully evaluated in future studies.

In summary, we have demonstrated unequivocally that PFOS-mediated Sertoli cell BTB disruption can be rescued by the use of an Akt activator SC79, which apparently exerts its effects by re-establishing the underlying actin- and MT-based cytoskeletons, restoring the localization of BTB-associated adhesion protein complexes. This thus reseals the disrupted Sertoli cell TJ-permeability barrier induced by PFOS. These findings should be carefully evaluated and further expanded to include the use of human Sertoli cells to assess if an activation of p-Akt could rescue PFOS-mediated Sertoli cell injury since a recent report has shown that Sertoli cell injury induced by environmental toxicants is similar to Sertoli cells isolated from rodent testes^46^. This additional study should be of important to therapeutically manage PFOS (or other environmental toxicants) induced testis injury and possibly infertility.

## Materials and Methods

### Animals and antibodies

Male Sprague-Dawley pups at 16–19 days of age, with a foster mother per 10 pups, were purchased from Charles River Laboratories (Kingston, NY). Animals were housed at the Rockefeller University Comparative Bioscience Center (CBC) with 10 pups and a foster mother per cage. All animals had free access to standard rat chow and water *ad libitum* under controlled temperature (22 °C) and constant light-dark cycles (12 hr of light: 12 hr of darkness). These animals were maintained in accordance with the applicable portions of the Animal Welfare Act and the guidelines in the Department of Health and Human Services publication *Guide for the Care and Use of Laboratory Animals*. The use of rats for experiments reported herein was approved by the Rockefeller University Institutional Animal Care and Use Committee (IACUC) with Protocol Numbers 12–506 and 15–780-H. All methods and experimental protocols used for relevant studies reported herein, including the use of animals and primary Sertoli cell cultures were carried out in accordance with the relevant guidelines, including any relevant details, and approved by the Rockefeller University Laboratory Safety and Environmental Health, the Rockefeller University Institutional Biosafety Committee (IBC), and the Rockefeller University Comparative Bioscience Center (CBC). These methods were also described in details in the sections below. Antibodies were obtained commercially and listed in Table 1.

### Primary Sertoli cell cultures

Sertoli cells were isolated from the testes of 20-day-old rats as earlier described^19^. Cells were seeded on Matrigel (1:5–1:7, diluted in F12/DMEM; BD Biosciences, San Jose, CA)-coated Millipore Millicell HA bicameral units (12-mm diameter, 0.6-cm^2^ effective surface area; 0.45-µm pore size; each unit was placed on a well of a 24-well dish containing 0.5 ml F12/DMEM in the apical and basal compartment), 6-well culture dishes (with each well contained 2 ml F12/DMEM to be used for lysate preparation) or coverslips (with each coverslip place on the well of a 12-well dish containing 1 ml F12/DMEM for immunofluorescence (IF) analysis) at densities of 1, 0.1 and ~0.03–0.04 × 10^6^ cells/cm^2^, respectively. Sertoli cells were cultured in serum-free F12/DMEM (Thermo Fisher Scientific, Waltham, MA) supplemented with growth factors and gentamicin and incubated in a humidified atmosphere of 95% air/5% CO_2_ (v/v) at 35 °C as described^19^. On day 2, Sertoli cells were subjected to a brief hypotonic treatment with 20 mM Tris (pH 7.4) for 2.5 min to lyse residual germ cells^74^, and replaced with fresh medium containing antibiotics and supplements. These Sertoli cell cultures contained ~98% Sertoli cells, with negligible contaminations of Leydig cells, peritubular myoid cells, and/or germ cells when specific markers of these cell types were used to assess their contamination by either immunoblotting (IB) or RT-PCR as described^75^. Sertoli cells cultured *in vitro* were shown to establish a functional TJ-permeability barrier with ultrastructures of TJs, basal ESs, gap junctions, and desmosomes that mimic the Sertoli cell BTB *in vivo* as described^21, 52^.

### Treatment of Sertoli cells with perfluorooctanesulfonate (PFOS) and SC79

Perfluorooctanesulfonate (PFOS) was obtained from Sigma-Aldrich (St. Louis, MO) and dissolved in DMSO at 100 mM. SC79 (2-amino-6-chloro-α-cyano-3-(eethoxycarbonyl)-4H-1-benzopyran-4-acetic acid ethyl ester, Mr 364.78), a specific Akt1/2 activator that binds to the plecktrin homology (PH) domain of Akt, mimicking the binding of PtdIns(3,4,5)P3 to induce Akt conformational change that enhances phosphorylation and activation at both the p-Akt1-T308 and p-Akt1-S473 sites^39^, was purchased from Millipore (Billerica, MA). SC79 was freshly prepared at 25 μg/μl in DMSO. Sertoli cells cultured for 3 days was treated with DMSO (control) *vs*. 10, 20 or 50 μM PFOS for 24 hr. For the rescue experiments, Sertoli cells were pre-treated with 2 μg/ml SC79 (5.5 µM) for 30 min as earlier described^39^, and then cells were rinsed and treated with PFOS (20 μM for IF; 50 μM for IB) for 24 hr. The same amount of DMSO was used in controls. The approach of pretreating cells with SC79 to examine its effects on Akt signaling was similar to earlier reports^39, 76^. However, in the experiments where the effects of SC79 to attenuate PFOS-mediated Sertoli cell TJ-barrier disruption were investigated, besides pre-treating cells with SC79 for 30 min, SC79 was also included during treatment of cells with PFOS for 24 hr. The selected concentrations of PFOS used in our experiments were based on earlier studies^13, 23, 77^, and were earlier shown to have no detectable cytotoxicity in Sertoli cells^13^. The concentration of SC79 used in our studies was similar to earlier studies on mammalian cells and tissues both *in vitro* and *in vivo* without cytotoxicity^39, 78^.

### Lysate preparation and immunoblotting (IB)

Protein lysates from primary Sertoli cells were obtained using IP lysis buffer [50 mM Tris, 0.15 M NaCl, 1% NP-40, 2 mM EGTA, and 10% glycerol (v/v), pH 7.4 at 22 °C] supplemented with protease phosphatase inhibitor mixture (Sigma-Aldrich) and phosphatase inhibitor mixture II (Sigma-Aldrich). Protein concentration was determined using a DC Protein Assay Kit from Bio-Rad (Hercules, CA). About 25–40 µg protein was used from each sample for immunoblotting. In short, equal amount of protein lysate from all samples within an experiment were resolved by SDS-PAGE and transferred onto nitrocellulose membrane (Bio-Rad) for immunoblot analysis using the corresponding primary and secondary antibody for a target protein (Table 1). Each target protein was visualized by enhanced chemiluminescence using a kit prepared in our laboratory as described^79^. Digital images were acquired and quantified using a Fujifilm LAS-4000 mini-Luminiscent Image Analyzer and Multi Gauge software package (Version 3.1) from Fujifilm Corp (Valhalla, NY) as earlier reported^35^. Due to space limitation to display multiple immunoblots, blots were cropped to illustrate only the target proteins to compare changes between treatment and control groups, and full-length blots for blots reported in Fig. 1B and Fig. 3A are shown in Figures S4 and S5, respectively, in Supplementary Information.

### Immunofluorescence (IF) analysis

IF was performed using Sertoli cells cultured on coverslips at a density of ~0.03–0.04 × 10^6^ cells/cm^2^. Sertoli cells were fixed in 4% PFA in PBS (10 mM sodium phosphate, 0.15 M NaCl, pH 7.4 at 22 °C) or ice-cold methanol, permeabilized with 0.1% Triton X-100 in PBS, and subsequently blocked with 1% BSA in PBS. Cells were then incubated with the corresponding primary antibodies (Table 1) at 4 °C overnight, to be followed by Alexa Fluor 488 (green) secondary antibodies (Thermo Fisher Scientific) for 1 hr. To visualize F-actin, sections and/or cells were incubated with FITC-conjugated phalloidin (Thermo Fisher Scientific) at 1:50 dilution for 1 hr. Cells on microscopic slides were mounted in Prolong Gold Antifade reagent with 4′, 6-diamidino-2-phenylindole (DAPI, Life Technologies) to visualize cell nuclei. Fluorescence images were examined and acquired using a Nikon Eclipse 90i Fluorescence Microscope system equipped with a Nikon Ds-Qi1Mc and a Nikon DS-Fi1 digital cameras, using the Nikon NIS Elements Imaging Software (Nikon Instruments, Inc). Image files were then analyzed using Photoshop in Adobe Creative Suite (Version 3.0; San Jose, CA) for image overlay to assess protein co-localization. All Sertoli cells within an experimental group were processed simultaneously in a single experimental session to eliminate interexperimental variations and each experiment was repeated at least 3 times. To assess changes in protein expression and/or distribution following treatment with DMSO by immunofluorescence microscopy, fluorescence intensity at the Sertoli cell-cell interface of a target protein in control *vs*. treatment group (at two opposite ends of adjacent cells, i.e., 4 measurements, to obtain an average which was the mean fluorescence intensity) in *in vitro* experiments was quantified using ImageJ 1.45 software (NIH, Bethesda, MD; http://rsbweb.nih.gov/ij). For changes in protein localization near the Sertoli cell cortical zone, the distribution of fluorescence signals at the cell-cell interface was measured at the two opposite ends (i.e., 4 measurements) of the Sertoli cell nucleus, which was then averaged to obtain the mean width. At least 200 cells were randomly selected and examined in control *vs*. experimental groups with *n* = 3 experiments (i.e., ~70 randomly selected cells per experiment).

### Monitoring the Sertoli cell TJ-permeability barrier function *in vitro*

Sertoli cells were plated on Matrigel (1:5)-coated bicameral units at 1 × 10^6^ cells/cm^2^. Bicameral units were then placed in 24-well plates with 0.5 ml F12/DMEM each in the apical and basal compartment. To assess the TJ-permeability barrier, both the PFOS and the control (DMSO) group contained triplicate bicameral units, and TER (transepithelial electrical resistance) across the cell epithelium was recorded daily. It is noted that TER monitored the ability of the Sertoli cell epithelium on the bicameral unit to resist conductivity of an electrical current that was sent across the electrodes of the Millipore Millicell ERS system as described^19^. Blanks contained Matrigel-coated bicameral units without Sertoli cells. TER experiments were repeated at least 3 times using different batches of Sertoli cells and yielded similar results.

### Statistical analysis

For studies using Sertoli cell cultures, triplicate coverslips, wells, or bicameral units were used for each experiment including treatment and control groups. Each data point (or bar graph) is a mean ± SD of *n* = 3 to 5 experiments. Statistical analysis was performed by one-way ANOVA followed by Dunnett’s test or nonparametric Mann-Whitney *U* test. In selected experiments, Student’s *t-*test was used for paired comparisons. Statistical analysis was performed using the GB-STAT software package (Version 7.0; Dynamic Microsystems, Silver Spring, MD). Mann-Whitney *U* test was performed using GraphPad Prism (Version 5.0, GraphPad Software, La Jolla, CA). *P* < 0.05 or smaller was considered statistically significant.

## Electronic supplementary material

Supplementary Information
