# Erratum: Perfluorooctanesulfonate (PFOS)-induced Sertoli cell injury through a disruption of F-actin and microtubule organization is mediated by Akt1/2

**DOI:** 10.1038/s41598-017-04863-7

**Published:** 2017-07-26

**Authors:** Ying Gao, Haiqi Chen, Xiang Xiao, Wing-yee Lui, Will M. Lee, Dolores D. Mruk, C. Yan Cheng

**Affiliations:** 1 The Mary M. Wohlford Laboratory for Male Contraceptive Research, Center for Biomedical Research, Population Council, 1230 York Ave, New York, New York, 10065 USA; 2 Department of Reproductive Physiology, Zhejiang Academy of Medical Sciences, Hangzhou, 310013 China; 3 School of Biological Sciences, The University of Hong Kong, Pokfulam, Hong Kong China

*Scientific Reports* **7**:1110; doi:10.1038/s41598-017-01016-8; Article published online 24 April 2017

The original version of this Article contained errors. In the Abstract,

“The use of SC79, an Akt1/2 inhibitor”

now reads:

“The use of SC79, an Akt1/2 activator”

In the Introduction,

“wildlife and animal exposure including fetal growth”

now reads:

“wildlife and animal exposure including reduced fetal growth”

In addition,

“inducing Sertoli cell injury and Leydig cell steroidogenic function”

now reads:

“inducing Sertoli cell injury and disrupting Leydig cell steroidogenic function”

Finally,

“This mechanistic study thus provides additional insight on the molecular mechanism by which PFOS mediates reproductive”

now reads:

“This mechanistic study thus provides additional insight on the molecular mechanism by which PFOS causes reproductive”

The original version of this Article omitted Xiang Xiao and Wing-yee Lui as corresponding authors. Correspondence and request for materials should also be addressed to X.X. (xxiao@zjams.com) or W.Y.L. (wylui@hku.hk)

In the Results section, under the sub-heading ‘PFOS perturbs Sertoli cell TJ-barrier function through changes in the spatial expression of actin binding and regulatory proteins’,

“Palladin also no longer stretched across the Sertoli cell cytosol to organize actin filaments into bundles, instead, paladin retracted from cell cytosol and found closer to the cell nuclei (Fig. 2).”

now reads:

“Palladin also no longer stretched across the Sertoli cell cytosol to organize actin filaments into bundles as seen in controls, instead, palladin retracted from cell cytosol and found closer to the cell nuclei following PFOS treatment (Fig. 2)”

The Results section contains an error in a sub-heading, where:

“Rescue of PFOS-induced Sertoli cell TJ-barrier disruption by SC79, an activator of Akt through changes in the re-distribution of BTB-associated proteins.”

now reads:

“Rescue of PFOS-induced Sertoli cell TJ-barrier disruption by SC79, an activator of Akts through changes in the re-distribution of BTB-associated proteins.”

There were also errors in the Figure legends. In Figure 2 legend,

“Instead, palladin was retraced from the cell peripheries but concentrated to cell nuclei after PFOS treatment.”

now reads:

“Instead, palladin was retracted from the cell peripheries but concentrated to cell nuclei after PFOS treatment.”

In Figure 3(c) legend

“(C) Sertoli cells cultured at 0.04 × 10^6^ cells/cm^2^ for 3 days were pre-treated with 2 μg/ml SC79 for 30 min, and then cells were rinsed and treated with 20 μM PFOS for 24 hr. Thereafter, cells were then fixed and examined by immunofluorescence microscopy. SC79 treatment was found to block PFOSinduced mis-localization of TJ proteins CAR and ZO-1, as well as basal ES proteins N-cadherin and β-catenin, apparently through enhanced endocytosis. BTB-associated proteins at the Sertoli cell-cell interface in control and treated cells were annotated by corresponding white and yellow brackets, respectively”

now reads:

“(C) Sertoli cells cultured at 0.04 × 10^6^ cells/cm^2^ for 3 days were pre-treated with 2 μg/ml SC79 for 30 min, and then cells were rinsed and treated with 20 μM PFOS for 24 hr. Thereafter, cells were then fixed and examined by immunofluorescence microscopy. SC79 treatment was found to block PFOS-induced mis-localization of TJ proteins CAR and ZO-1, as well as basal ES proteins N-cadherin and β-catenin. BTB-associated proteins at the Sertoli cell-cell interface in control and PFOS-treated cells were annotated by corresponding white and yellow brackets, respectively”

In Figure 4 legend

“yellow rectangles in FOS-treated cells”

now reads:

“yellow rectangles in PFOS-treated cells”

In Figure 5 legend, Figure 1A now reads Figure 1B

In Figure 6 legend,

“Thereafter, cells were fixed with ice-cold methanol and visualized byimmunofluorescence microscopy.”

now reads:

“Thereafter, cells were fixed with ice-cold methanol and visualized by immunofluorescence microscopy using corresponding antibodies (Table 1)”

In addition, the Acknowledgments section now reads:

We wish to thank Dr. Ben Margolis and Dr. Shuling Fan who kindly provided us with some anti-CRB3 antibody to validate some experiments in this report. This work was supported by grants from the National Institutes of Health (R01 HD056034 to C.Y.C.; U54 HD029990 Project 5 to C.Y.C.); National Natural Science Foundation of China (NSFC) (31371176 to X.X.), Qianjiang Talents Program (QJD1502029 to X.X.), Zhejiang Province Funding (Science Technology Department, 2016F10010 to X.X.); Hong Kong Research Grants Council (RGC) General Research Fund (GRF) (774213 and 17100816 to W.Y.L.; 771513 to W.M.L.), NSFC/RGC Joint Research Scheme (N_HKU 717/12 to W.M.L.), and University of Hong Kong CRCG Seed Funding (to W.M.L.)

The Author Contributions statement now reads:

C.Y.C., Y.G. and H.C. designed research; Y.G., H.C. and C.Y.C. performed research; X.X., W.Y.L., W.M.L., D.D.M. and C.Y.C. contributed new reagents/analytic tools; Y.G., H.C. and C.Y.C. analyzed data; Y.G. and C.Y.C. wrote the paper; Y.G., H.C. and C.Y.C. prepared all figures. All authors reviewed the manuscript.

There were also errors in References 43 and 76, which now read:

43. Li, N., Mruk, D. D., Lee, W. M., Wong, C. K. C. & Cheng, C. Y. Is toxicant-induced Sertoli cell injury *in vitro* a useful model to study molecular mechanisms in spermatogenesis? *Semin Cell Dev Biol* **59**, 141–156, doi:10.1016/j.semcdb.2016.01.003 (2016).

76. Gong, Y. *et al*. SC79 protects retinal pigment epithelium cells from UV radiation via activating Akt-Nrf2 signaling. *Oncotarget* **7**, 60123–60132, doi:10.18632/oncotarget.11164 (2016).

These errors have now been corrected in the HTML and PDF versions of this Article.

