# Supplementary material for: Perfluorooctanesulfonate (PFOS)-induced Sertoli cell injury through a disruption of F-actin and microtubule organization is mediated by Akt1/2
Source: Sci Rep. 2017 Apr 24;7:1110. doi: 10.1038/s41598-017-01016-8 (PMC5430865; doi:10.1038/s41598-017-01016-8)

Supplementary Information

Perfluorooctanesulfonate (PFOS)-induced Sertoli cell injury through a disruption of F-actin and microtubule organization is mediated by Akt1/2

Ying Gao, Haiqi Chen, Xiang Xiao, Wing-yee Lui, Will M. Lee, Dolores D. Mruk and C. Yan Cheng

Figure S1. Effects of PFOS on the steady-state levels and distribution of proteins at the Sertoli cell BTB. On day 3, Sertoli cell cultured alone with the establishment of a functional TJ-permeability barrier were treated with PFOS at 10, 20 or 50 µM *vs.* controls (vehicle alone, i.e., DMSO without PFOS) for 24 hr. Cells were terminated on day 4 and used for either immunoblotting (IB) or IF as shown in Figure 1. (A) Composite data from immunoblots, normalized against ß-actin used for protein loading control, such as those shown in Figure 1B, with each bar a mean±SD of *n* = 3 independent experiments. For immunoblot data for p-Akt1-T308, p-Akt1-S473 and p-Akt2-S474 and also for p-FAK-Y407, these target proteins were also normalized again the total Akts and FAK. The level of a target protein in control (DMOS vehicle control alone with PFOS) was arbitrarily set at 1 against which statistical comparison was performed. *, $P<0.05$; **, $P<0.01$; by Mann-Whitney *U* test. (B) Image analysis was performed in which the fluorescence intensity of occludin and CAR at the Sertoli cell-cell interface was assessed in control (Ctrl) cells (see white arrowheads) *vs.* PFOS-treated cells at either 20 or 50 µM (see yellow arrowheads) in Figure 1C. Each bar is a mean±SD of 200 Sertoli cells from *n* = 3 experiments (~70 randomly selected cells in each experiment). For ZO-1, N-cadherin and ß-catenin, changes in protein distribution was assessed in treatment group (see yellow brackets) *vs.* control cells (see white brackets) at the Sertoli cell-cell interface. Each bar is also a mean±SD of 200 Sertoli cells from *n* = 3 experiments (see *Materials and Methods*). *, $P<0.05$; **, $P<0.01$; by ANOVA.

Figure S2. Effects of SC79, an activator of Akts, on restoring PFOS-mediated down-regulation of p-Akt1 and disruptive changes in the localization of BTB-associated proteins. Experimental conditions are shown in the legend to Figure 3. (A) Composite data from immunoblots, normalized against ß-actin used for protein loading control, such as those shown in Figure 3A. For immunoblot data for p-Akt1-T308, p-Akt1-S473 and p-Akt2-S474, these target proteins were also normalized again the total Akts. Each bar is a mean±SD of *n* = 3 independent experiments. The level of a target protein in control (DMOS vehicle control alone with PFOS) was arbitrarily set at 1 against which statistical comparison was performed. SC79 was found to rescue the PFOS-mediated down-regulation of p-Akt1-S473 but not p-Akt2-S474. *, $P<0.05$ by ANOVA followed by Dunnett's test. (B) Image analysis was performed to assess changes in the protein distribution of a target protein such as CAR, ZO-1, N-cadherin and ß-catenin following treatment (see yellow brackets) *vs.* control (Ctrl) (see white brackets) at the Sertoli cell-cell interface. Each bar is also a mean±SD of 200 Sertoli cells from *n* = 3 experiments. **, $P<0.01$ by ANOVA followed by Dunnett's test.

Figure S3. Effects of SC79 on restoring PFOS-mediated disruption on the distribution of Arp3 and p-FAK-Y407. Experimental conditions are shown in the legend to Figure 4. Composite data of image analysis of *n* = 3 experiments with ~70 randomly selected Sertoli cells were analyzed in which changes on the distribution of either Arp3 or p-FAK-Y407 at the Sertoli cell cortical zone was quantified by measuring the fluorescence intensity of each target protein in control cells (see dotted while rectangles) *vs.* PFOS-treated cells (see dotted yellow rectangles). See legend to Figure 4 for details. **, $P<0.01$, by ANOVA followed by Dunnett's test.

Figure S4. Immunoblots that illustrate the effects of PFOS on the expression of selected BTB-associated proteins. These are uncropped blot images corresponding to data shown in Figure 1B.

Figure S5. Immunoblots that illustrate the ability of Akt activator SC79 to block the effects of PFOS to down-regulate the expression of selected BTB-associated proteins. These are uncropped blot images corresponding to data shown in Figure 3A.

Figure S1 (Gao et al.)

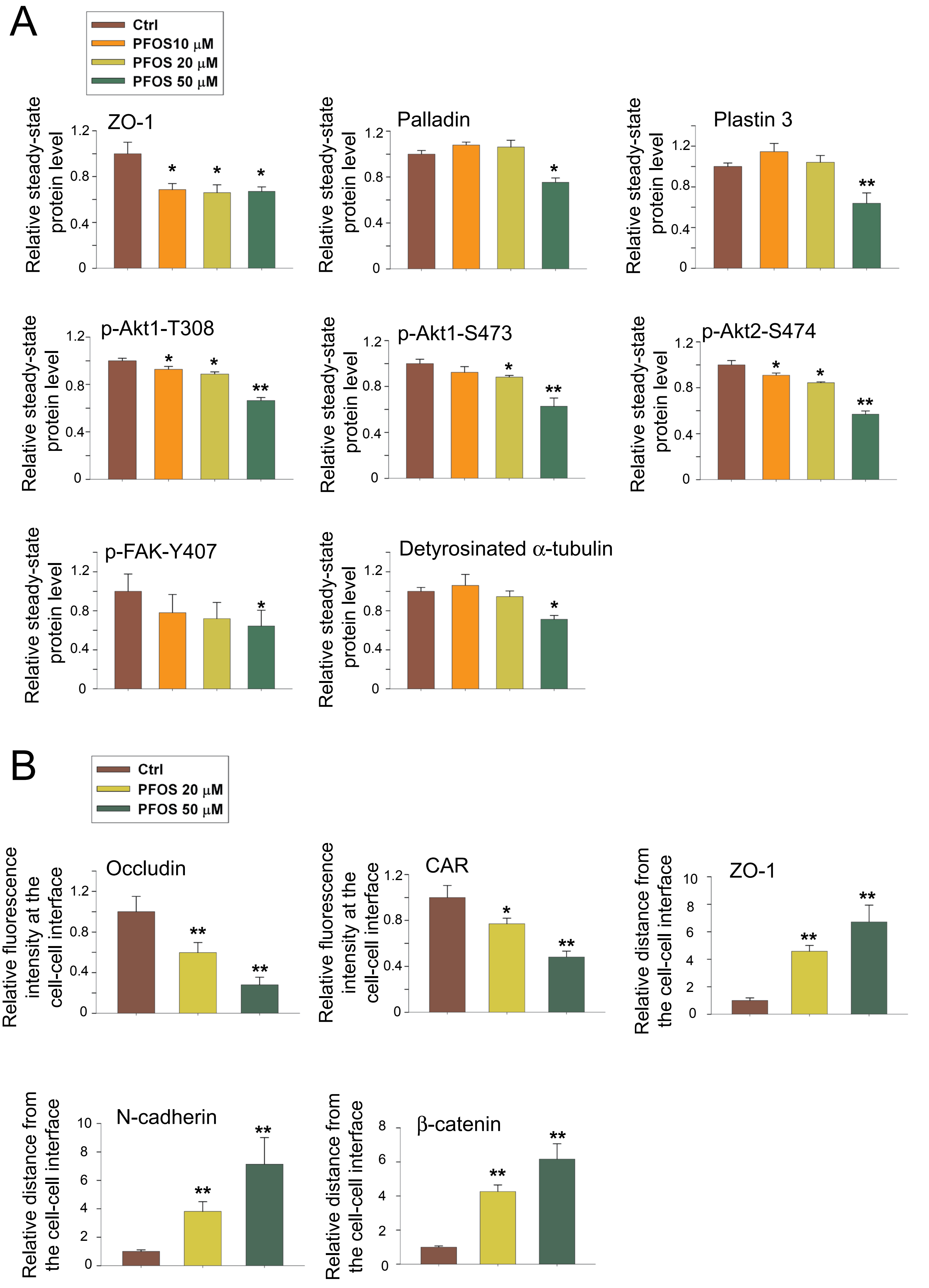

Figure S2 (Gao et al.)

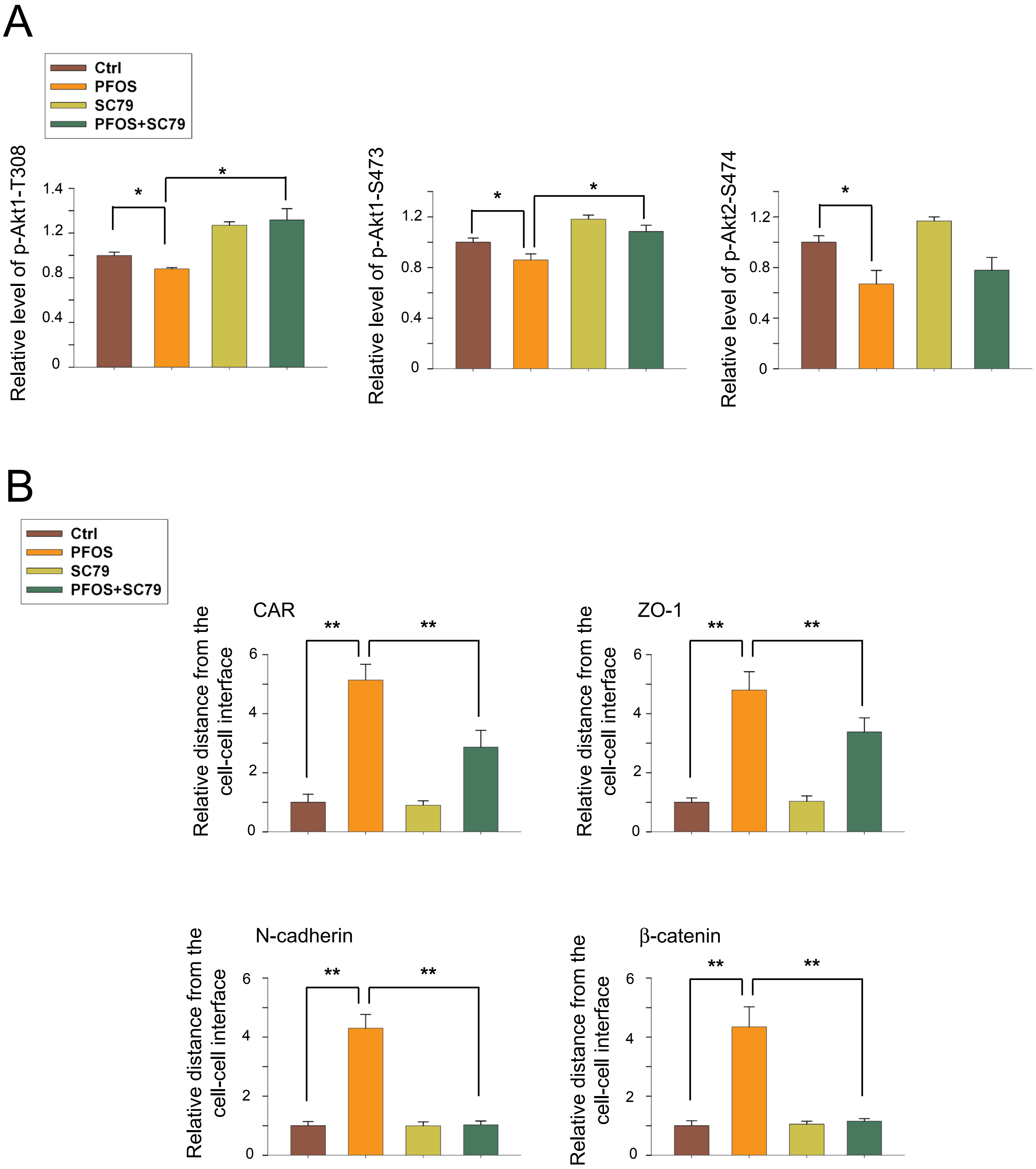

Figure S3 (Gao et al.)

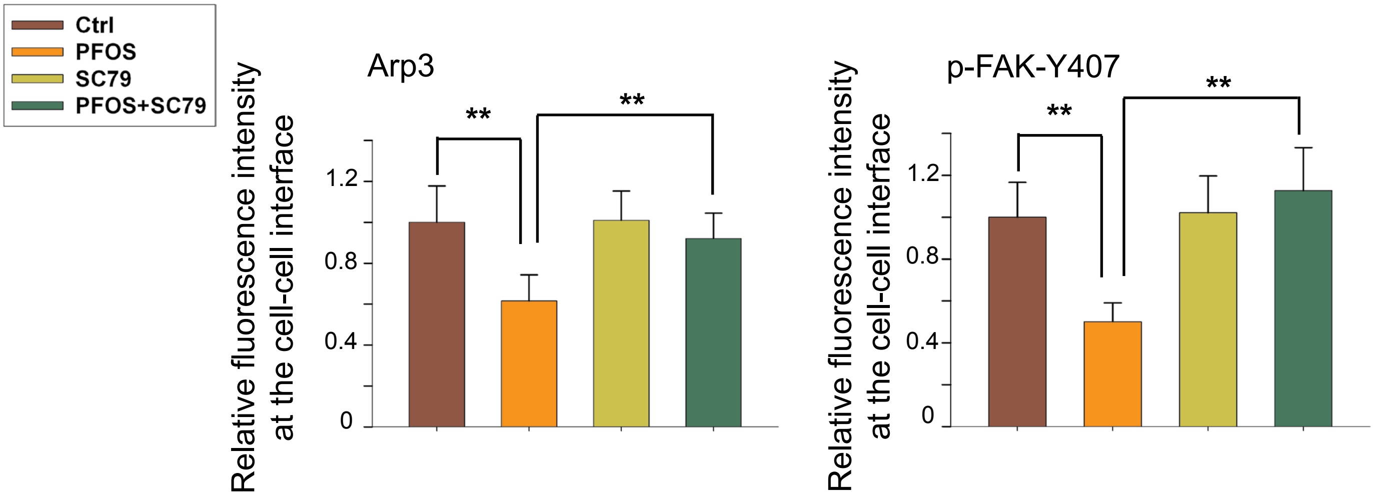

Figure S4 (Gao et al.)

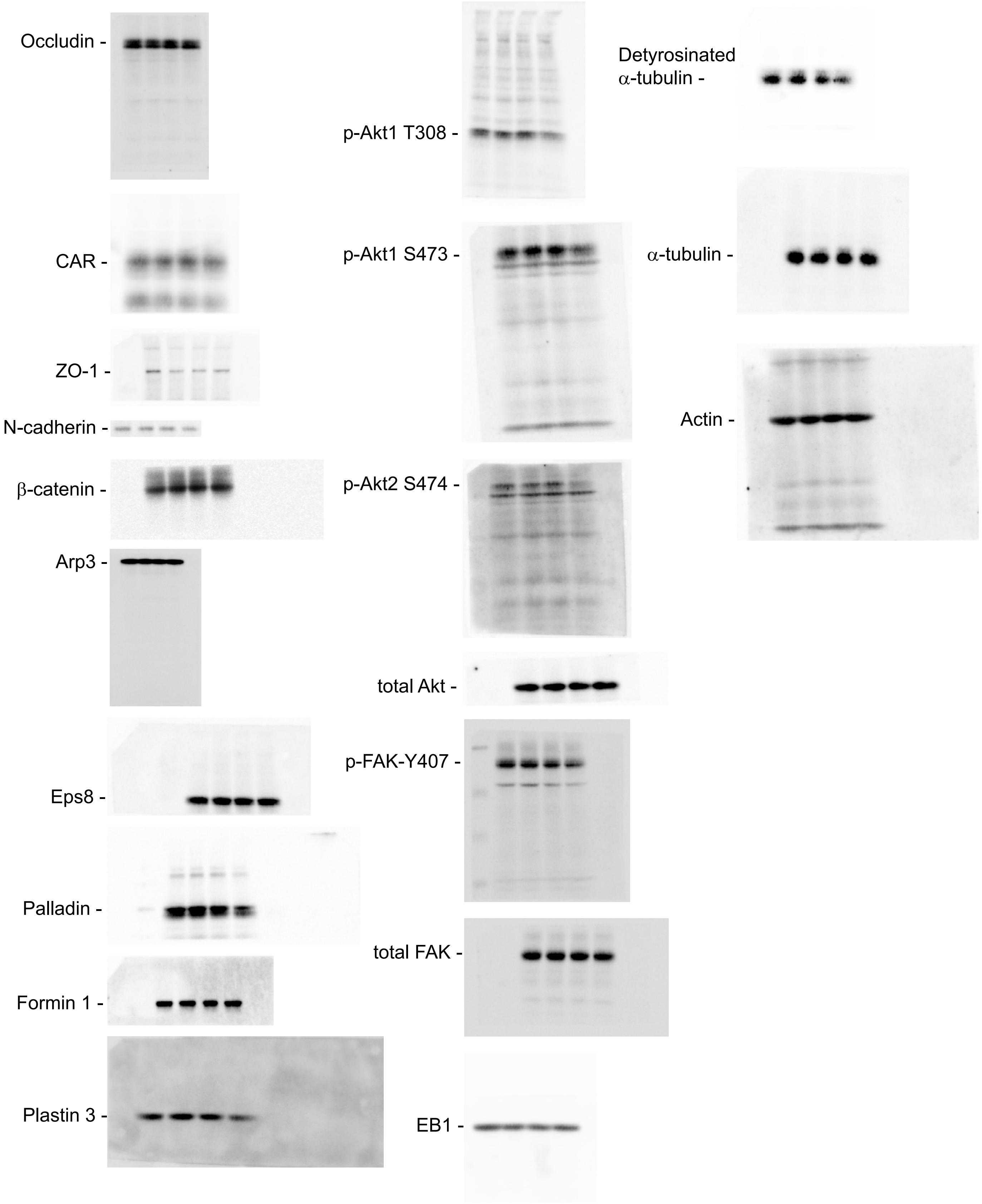

Figure S5 (Gao et al.)

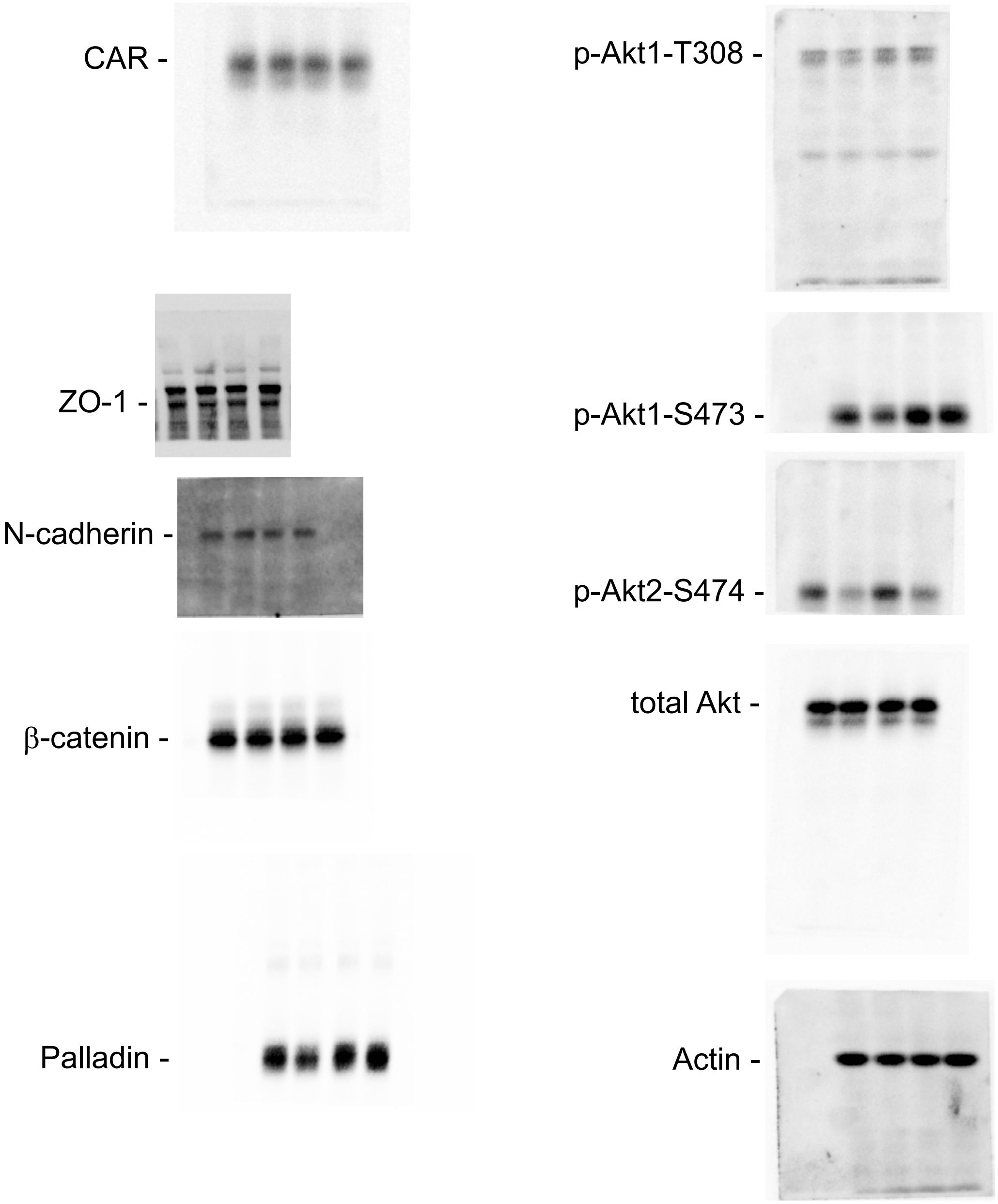

Supplement: Supplementary file 1 — Supplementary Information [file 41598_2017_1016_MOESM1_ESM.pdf]
